# International survey on diagnostic reference levels based on clinical indications in plain radiography

**DOI:** 10.1007/s00330-024-11224-2

**Published:** 2024-12-04

**Authors:** Alexander A. Schegerer, Georg Stamm, Christoph Aberle, Josefin Ammon, Babak Bazrafshan, Markus Borowski, Rainer Eßeling, Bärbel Madsack, Roman Menz, Constance Müller, Nadia Oberhofer, Bernhard Renger, Julian Singer, Michael Verius, Michael Walz, Kerstin Jungnickel

**Affiliations:** 1 Department of Radiation Protection and Radiological Processes, Hirslanden Hospitals, Corporate Office, Boulevard Lilienthal 2, 8152 Opfikon-Glattpark, Switzerland; 2 MPS Medical Physics and Radiation Protection GmbH, Breitenfeldstrasse 46, 91126 Schwabach, Germany; 3 Institute for Clinical and Interventional Radiology, University Medical Center Göttingen, Robert-Koch-Strasse 40, 37075 Göttingen, Germany; 4 Clinic of Radiology and Nuclear Medicine, University Hospital Basel, University of Basel, Petersgraben 4, 4031 Basel, Switzerland; 5 Institute of Medical Physics, Nuremberg General Hospital, Paracelsus Medical University, Prof.-Ernst-Nathan-Strasse 1, 90419 Nuremberg, Germany; 6 Radiation Protection and Laser Safety Unit, University Hospital Tübingen, Auf der Morgenstelle 24, 72076 Tübingen, Germany; 7 Institute of Radiology and Nuclear Medicine, Municipal Hospital Braunschweig, Freisestrasse 9/10, 38118 Braunschweig, Germany; 8 Clinic for Radiology, University Hospital Münster, Albert-Schweitzer-Campus 1, 48149 Münster, Germany; 9 Medical Office for Quality Assurance in Radiology, Nuclear Medicine and Radiation Therapy (Ärztliche Stelle Hessen), TÜV SÜD Life Service GmbH, Am Römerhof 15, 60486 Frankfurt, Germany; 10 Azienda Sanitaria dell’Alto Adige, Servizio Aziendale di Fisica Sanitaria, via Lorenz Böhler 5, 39100 Bolzano, Italy; 11 Department of Diagnostic and Interventional Radiology, School of Medicine & Health, Klinikum Rechts der Isar, Technical University of Munich, Ismaninger Strasse 22, 81675 Munich, Germany; 12 Department of Radiology, Medical University Innsbruck, Anichstraße 35, 6020 Innsbruck, Austria; 13 Institute of Diagnostic and Interventional Radiology, Municipal Hospital Magdeburg, Birkenallee 34, 39130 Magdeburg, Germany

**Keywords:** Radiography, Radiation dosage, Diagnostic reference levels, Radiation protection, Europe

## Abstract

**Purpose:**

To collect and analyze radiation dose-related data as part of international cooperation; to define diagnostic reference levels (DRL) for 24 X-ray projections in plain radiography (DX) considering anatomical region, clinical task, and procedural technique; and to harmonize the exposure practice across country borders.

**Methods:**

A multicenter study was performed in Austria, Germany, Italy, and Switzerland in 2022–23 to provide dose-related data. Healthcare facilities were asked to provide processed data from their dose management systems. A 5%-level was used for assessing the statistical significance of dose differences between various groups.

**Results:**

Dose-related data from 85 radiographic systems in academic and non-academic, public, and private healthcare facilities were analyzed. Dose-related parameters differed significantly for many projections with different clinical tasks and techniques. Dose-related data of the procedures varied by a maximum factor of 16 for the same system, and median dose values also differed between hospitals by a maximum factor of 31. A fifth of the surveyed systems exhibit doses above more than half of the new DRLs defined in this study. Apart from the three reference procedures, no significant dose differences were observed between X-ray systems of different ages, from different manufacturers, or from different countries.

**Conclusions:**

This is the first survey in which exposure practices were investigated in institutions in different central European countries by establishing clinical DRLs for radiography. The observed dose variations could be explained by different reasons, such as non-optimized dose protocols. The new DRLs help to harmonize the exposure practice across country borders.

**Key Points:**

***Question*** *What is the exposure practice for plain radiography procedures for which no clinical diagnostic reference levels (DRLs) have been defined? Are there differences between countries?*

***Findings*** *The dose for the same clinical task and technique can vary considerably among institutions but, on average, do not significantly differ between neighboring countries in Europe.*

***Clinical relevance*** *In this international multicenter study, clinical DRLs were defined for 24 plain radiography projections to promote the optimization of the exposure practice, to reduce dose variations among institutions even across national borders, and to strengthen international cooperation among users.*

## Introduction

All users of ionizing radiation in medical procedures, such as physicians or radiographers, take clinical responsibility for limiting radiation to only medically justified procedures and reducing radiation dose to a level that is as low as reasonably achievable (ALARA). Furthermore, clinical responsibility includes cooperating with radiation protection specialists, such as medical physicists, to get adequate support in radiation protection. The assumption of clinical responsibility applies to all X-ray procedures regardless of their dose ranges [1, 2].

Various guidelines support users in their selection of the most medically indicated imaging procedure for a specific clinical task [3–5]. ALARA does not mean dose reduction. The purpose of ALARA is rather to optimize radiation protection, i.e., to adapt radiation exposure to the clinical task, procedural technique, complexity level of the procedure, anatomical location, and patient size. To promote optimization, the European Basic Safety Standards Directive asks all European member states to establish, implement, and regularly review diagnostic reference levels (DRLs) for X-ray procedures. According to the International Commission on Radiological Protection (ICRP), DRL is the Commission’s term for a form of investigation level: users should investigate whenever DRLs are continuously exceeded and implement measures to achieve compliance with DRLs [6]. Numerically, a DRL is defined as the 3rd quartile value of the distribution of median values of a dose quantity acquired at various healthcare facilities (e.g., kerma-area product, *P*_KA_, also known as dose area product). Until recent years, DRLs were mostly specified for anatomical locations (anatomical DRLs, e.g., for spine) [7]. But in addition to the anatomical location, the clinical task (e.g., suspected scoliosis) and procedural technique (e.g., stitched anterior-posterior (AP) views) were recommended to be taken into account for an unequivocal definition of a DRL and, thus, for a more efficient optimization of a procedure [6, 8]. In this context, the term clinical DRL was introduced to underline the intention to define DRLs not only for an anatomical location but also for a specified clinical task and procedural technique. Based on this CAP approach (clinical task, anatomical location, procedural technique), clinical DRLs have been defined for various procedures in CT [8, 9] and interventional radiography (IR) [10, 11]. For plain radiography (DX), only very few clinical DRLs have been defined so far, e.g., the Norwegian DRL for the DX procedure of the pelvis after prosthesis surgery [8, 12] or a clinical DRL for the DX procedure for dysplasia of the hip in children [13].

To comply with the recommendations of different stakeholder organizations, such as the German Commission on Radiation Protection and the Federal Office for Radiation Protection, the Roentgen Society’s Working Group of Physics and Technology (APT) launched a survey to acquire dose-related data and to define DRLs for various frequently performed DX, IR, and CT procedures for which national DRLs do not yet exist. In order to get a larger database, data were acquired from various institutions in Austria, Germany, Italy, and Switzerland. Due to previous individual cooperations, it was already known that technical equipment and protocol parameters do not significantly differ between the countries and that it is, therefore, possible to define common DRLs.

The aim of this paper is to report the methods and results of the survey on DX procedures and to propose clinical DRLs. This international approach complies with a request of the European Commission (EC) to harmonize exposure practices across country borders [8].

## Methods and materials

In this study, only anonymous and summarized dose-related data (mean values, quartiles) were analyzed. We have received confirmation from institutional clinical trial units/ethical commissions that no national and international declarations and regulations for the protection of humans would be infringed by this study.

Based on the information of the study participants and the specification of the stakeholders, 24 DX projections were selected (hereafter referred to as “reference projections”). In most participating institutions, these projections were carried out much more than 20 times a year. In accordance with the CAP approach, the clinical question and the procedural technique were specified in addition to the anatomical region (Table 1). Experience from previous surveys served as a guideline concerning the format, content, and execution of this retrospective survey [8, 14–16].

**Table 1:** Definition of the clinical task, anatomical location, and procedural technique, including view of the reference projection surveyed in this international multicenter survey

	Clinical task	Anatomical location	Procedure technique, view
1	Traumatic or degenerative change	Cervical spine (C3–C7 for AP, C1–C7 for LAT-view)	AP LAT
2			
3	Traumatic change of dens axis, exclusion of atlanto-axial subluxation	Dens axis (C1–C3)	AP (with open mouth)
4	Non-traumatic foraminal stenosis, foraminal tumor or metastases, arthrotic change	Cervical spine (C1–C7)	OBL (ca. 45° LAO/RAO)
5	Traumatic or degenerative change, luxation of acromioclavicular joint	Clavicle	AP TAN (45° caudal-cranial)
6			
7	Traumatic or degenerative change	Elbow	AP LAT
8			
9	Traumatic or degenerative change	Wrist	DV LAT
10			
11	Traumatic or degenerative change, arthritis, foreign particles	Hand	DV LAT OBL
12			
13			
14	Traumatic change	Upper/lower ribs	AP OBL (ca. 45° LAO/RAO)
15			
16	Deformity of spine, scoliosis	Entire spine	AP, stitched exposure LAT, stitched exposure
17			
18	Traumatic and degenerative change	Knee	AP LAT
19			
20	Arthritic joint	Joint gap of knees	PA (Rosenberg projection)
21	Traumatic or degenerative change	Upper ankle joint	AP LAT
22			
23	Leg length discrepancy, measurement of leg axes	Entire leg (pelvis-foot)	AP, stitched exposure
24	Leg length discrepancy, measurement of leg axes	Entire leg (both legs)	AP, stitched exposure

*C1* cervical vertebral body no. 1, *AP* anterior-posterior, *PA* posterior-anterior, *LAT* lateral, *TAN* tangential, *LAO* left anterior oblique, *RAO* right anterior oblique, *DV* dorsovolar

The participating facilities were the radiological departments of academic or non-academic, public or private hospitals of different size, as well as private practices. The contact persons of the facilities were very experienced in dose optimization, with more than 10 years of professional experience in this field. They were reported to take national DRLs into consideration during protocol design and to investigate reasons for any dose excess.

X-ray systems in the participating facilities are subject to periodic quality assurance controlled by local or national authorities. In accordance with the international standard [17], the deviation of the displayed from actual dose measurements must be less than 30%. To minimize the required time investment of the participants for this study, the processed output of dose management systems (DMS) was used. The proper functioning of the DMS was ensured by the contact persons who were responsible for monitoring the dose-related data of the systems included in this study [18]. Apart from Swiss facilities, *P*_KA_ was usually given in the physical unit centi-Gray-square centimeters, which was then used in this study.

The following information was requested:

1. The year of installation and model of the surveyed system.
2. The year of installation and the software company of the DMS. Participants were asked for the filter criteria used for the assignment (mapping) of individual radiographs to the reference projections.
3. The view of the radiographs: anterior-posterior (AP), lateral (LAT), oblique (OBL), or tangential (TAN).
4. For each of the reference procedures (Table 1), the quartiles and the mean of the *P*_KA_ and—if available—the median value of the tube voltage computed with the DMS from at least 10 radiographs of adult patients (age > 16 years old).
5. For (stitched) exposures that are combined over 2–3 adjacent body areas, quartiles and mean had to be computed from the sums of the *P*_KA_, which were formed from the *P*_KA_ of the single exposures.

During the survey, which was conducted in 2022–23, all collected data were reviewed to ensure that they were in the correct format and order of magnitude and correctly mapped to one of the reference projections. In cases where potentially incorrect information was observed (e.g., particularly high or low *P*_KA_), participants were contacted to verify, correct, or complete the data. Dose-relevant parameters that could not be correctly mapped to the reference procedures because of missing information on the view, for instance, were not included in the data analysis. Teleconferences among participants were organized to solve any issues that arose. Participants were finally informed about their local exposure levels compared to the DRLs defined in this study.

For each reference projection, the quartiles and mean were computed from the distribution of median values of *P*_KA_. The 3rd quartile was defined as DRL. The statistical significance in the non-normally distributed dose data from different procedures or different subgroups (e.g., countries) was tested using either the Mann-Whitney or the Kruskal–Wallis test, depending on how many subgroups were considered. All statistical tests were performed at a significance level of *p* < 0.05 with the program IDL (version 8.7.2., Harris Geospation Solutions).

## Results

Because of the call of the APT, an international network of medical physicists, radiographers and radiologists could be established for this study. Dose-related data were obtained from 2 Austrian, 35 German, 16 Italian and 32 Swiss DX systems in academic and non-academic, public and private healthcare facilities. For data acquisition, all common DMSs from the major manufacturers were represented in the study. One hospital used an in-house programmed DMS.

**Fig. 1:**
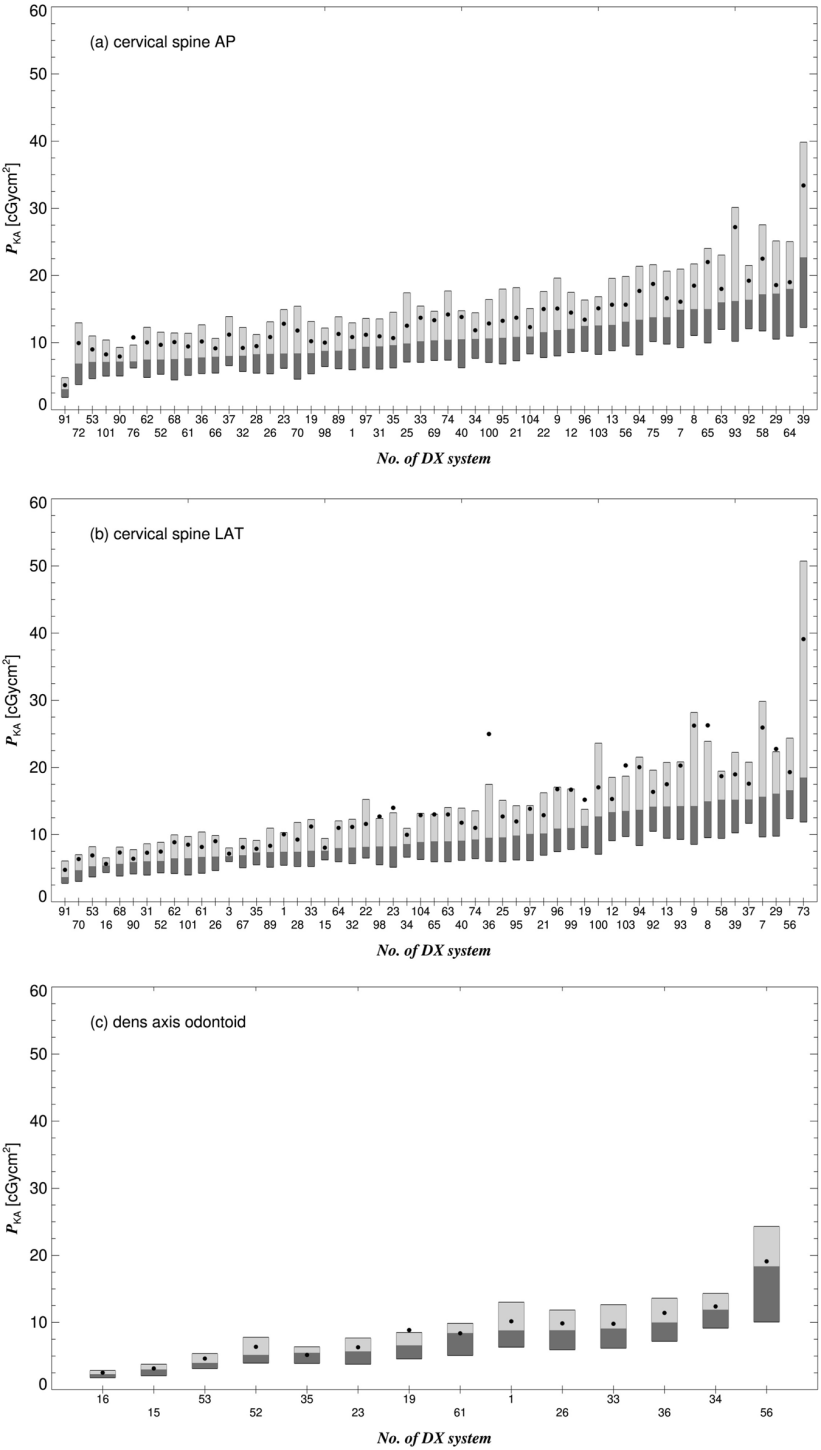
Box plots of the kerma-area product (*P*_KA_), shown for the radiographic (DX) systems (*x*-axis) that submitted dose-related data for the (**a**) AP- and (**b**) LAT-view of the cervical spine and for (**c**) the AP-view of the dens axis. The boxes are drawn around the 1st and 2nd (dark gray) as well as the 2nd and 3rd quartiles (light gray). Filled circles represent the mean

**Fig. 2:**
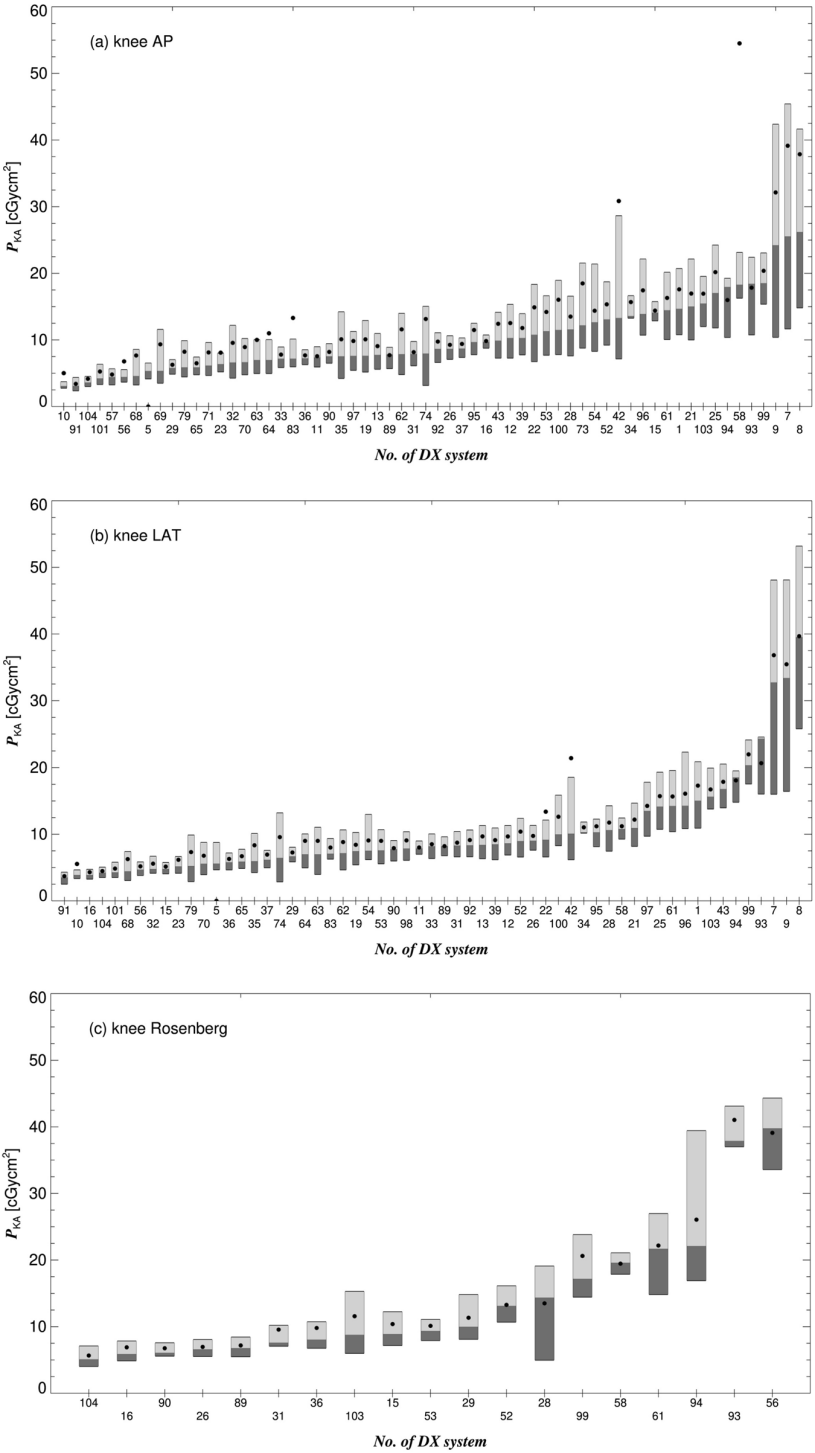
Box plots of the *P*_KA_, shown for the DX systems that submitted dose-related data for the (**a**) AP-, (**b**) LAT- and (**c**) «Rosenberg»-view of the knee. The boxes are drawn around the 1st and 2nd (dark gray) as well as the 2nd and 3rd quartiles (light gray). Filled circles represent the mean

**Fig. 3:**
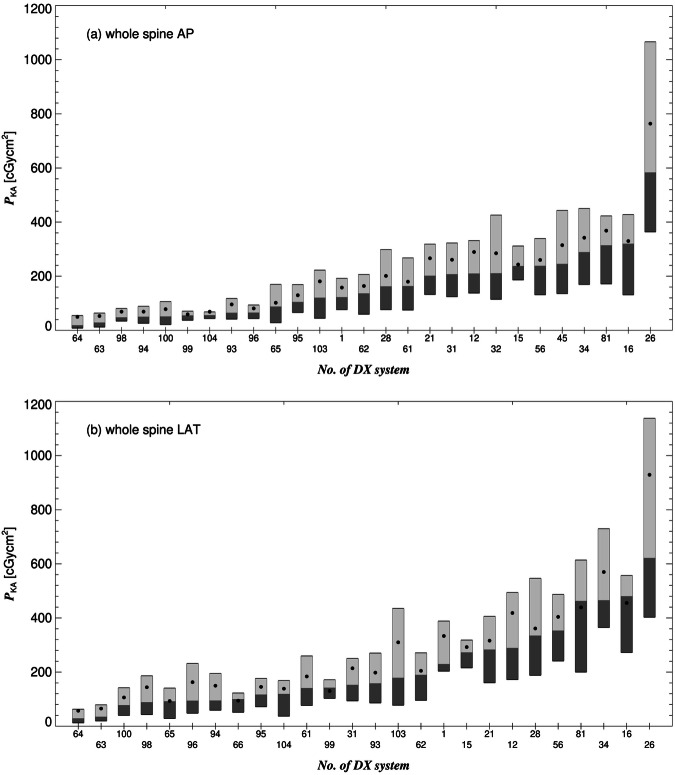
Box plots of the *P*_KA_, shown for the DX systems that submitted dose-related data for the (**a**) AP- and (**b**) LAT-view of the entire spine. The boxes are drawn around the 1st and 2nd (dark gray) as well as the 2nd and 3rd quartiles (light gray). Filled circles represent the mean

As the participating facilities specialized in different medical fields, they could not submit dose-related data for all the reference procedures equally. For instance, radiographs of the entire leg and entire spine were performed less frequently, mostly in specialized orthopedic hospitals. The number of systems that provided data for the corresponding reference procedures, the 1st, 2nd, and 3rd quartile, as well as the mean of the distribution of median values of *P*_KA_ and the 2nd quartile of median values of the tube voltage, are listed in Table 2. In Figs. 1–3, as examples, the positions of the quartiles and mean of *P*_KA_ are shown for different projections of the cervical spine, knee, and entire spine.

**Table 2:** Number of contributing X-ray systems, 1st quartile, 2nd quartile, 3rd quartile, mean of median values of *P*_KA_, and 2nd quartile of median values of the tube voltage for the 24 surveyed reference projections

	Reference projection	Number	1st quartile	2nd quartile	3rd quartile (DRL)	Mean	2nd quartile
			of median values of *P*_KA_ (cGycm)^2^				*U* (kV)
1	**Cervical spine AP**	55	8.1	10	**13**	11	66
2	**Cervical spine LAT**	55	7.0	9.0	**14**	9.9	70
3	**Dens axis odontoid**	14	5.2	7.5	**9.1**	7.7	70
4	**Cervical spine OBL**	18	5.7	7.9	**14**	9.0	70
5	**Clavicle AP**	34	5.6	11	**16**	12	63
6	**Clavicle TAN**	26	6.8	8.0	**13**	10	66
7	**Elbow AP**	41	1.7	2.4	**3.4**	2.4	55
8	**Elbow LAT**	40	2.0	2.4	**4.0**	2.7	55
9	**Wrist DV**	40	0.8	0.9	**1.2**	1.1	50
10	**Wrist LAT**	41	0.8	0.9	**1.4**	1.2	52
11	**Hand DV**	40	1.2	1.7	**2.2**	1.8	50
12	**Hand LAT**	19	0.9	1.2	**1.8**	1.3	50
13	**Hand OBL**	36	1.2	1.5	**2.0**	1.6	50
14	**Ribs AP**	30	27	36	**48**	39	73
15	**Ribs OBL**	20	35	45	**60**	48	68
16	**Entire spine AP**	27	58	137	**230**	160	77–86
17	**Entire spine LAT**	26	95	156	**290**	220	83–90
18	**Knee AP**	62	6.7	8.3	**13**	10	63
19	**Knee LAT**	59	5.9	8.2	**11**	10	63
20	**Knee Rosenberg**	19	7.0	9.4	**19**	14	64
21	**Upper ankle joint AP**	47	1.8	2.5	**3.4**	2.8	55
22	**Upper ankle joint LAT**	47	1.9	2.8	**3.8**	3.0	55
23	**Entire leg AP**	10	15	75	**110**	67	81–85
24	**Entire legs AP**	17	35	110	**180**	110	75–88

The 3rd quartile of median values of *P*_KA_ represents the corresponding DRLs (bold font). As the projections of the entire spine and legs consist of 2 to 3 (stitched) exposures, a range of tube voltages (2nd quartile of minima of tube voltages—2nd quartile of maxima of tube voltages) is given. For the exposure of the pelvis (for the entire spine) and of the upper thigh (for the entire leg), higher tube voltages are usually used than for the cervical part of the spine and for the lower legs, respectively

*P*_KA_ kerma-area product, *U* tube voltage

The interquartile ratio of the 3rd to the 1st quartile of an imaging system (the ratios of the upper and lower margins of the boxes shown in Figs. 1–3) is a measure for the *intra*-system dose variation, i.e., how strongly dose values are spread for the *same* system and the *same* reference projection. Particularly high intra-system dose discrepancies were found for clavicle TAN (16 at maximum) and the ribs AP (11). Averaged over all systems and reference projections, the intra-system dose discrepancy is 2.5.

For the same reference projection, the median values of *P*_KA_, are represented by the lines that divide the boxes in Figs. 1–3, can also strongly differ among the contributing systems, e.g., up to a factor 31 for the entire spine AP, showing large *inter*-system dose discrepancy. Two-thirds of the contributing systems do not comply with at least one of the 24 DRLs defined in this study (Table 2). A fifth exceeds more than half of these DRLs.

In Table 2, the 2nd quartile of median values of tube voltages is listed for each reference projection. They largely correspond to the specifications of a national recommendation [19]. The median voltages of the radiographs of the entire legs and entire spine (both views) vary between ca. 75 kV (lower leg or cervical part of the spine) and 90 kV (upper thigh or pelvis) with high *inter*-hospital variations within the *same* anatomical region of the legs.

For the radiographs of knees and entire legs, *P*_KA_ increases significantly when examining corresponding body sections of *both* extremities instead of one. There are statistically significant dose differences between the reference projections of the dens axis and other views of the cervical spine, between ribs AP and lateral (LAT), and hand DV and wrist DV.

All X-ray systems were equipped with flat panel detectors. On average, the systems were 7 years old in 2023, and 63% of the systems were produced by two manufacturers. No significant differences were found between *P*_KA_ values of different ages (older or younger than the average age) and different manufacturers.

For all reference projections, there is no significant dose difference between the surveyed German and Swiss systems. Between the German and Italian or Swiss and Italian systems, the doses differed significantly for the clavicle, upper ankle joint, and entire spine imaging procedures. The reason could be that the same medical physicist looks after all the surveyed (Northern) Italian systems resulting in a potential systematic dose bias. This physicist recently made intensive efforts to optimize the protocols of the radiographs of the entire spine, and this explains why the Italian dose values of this procedure were significantly (70%) smaller than the median dose values derived from the German and Swiss facilities, i.e., only a few German and Swiss systems underwent similarly intensive optimization efforts to date. The number of contributing Austrian facilities is too small to be considered in the assessment of the statistical significance. However, the level of their dose-related data is in good agreement with the data from other countries.

## Discussion

DRLs, in particular clinical DRLs, are an essential tool to objectively assess and harmonize the exposure practice and to identify procedures that may require optimization. According to numerous previous studies, the comparison of dose-relevant parameter values with the corresponding clinical DRLs has led to improved the adaption of protocol parameters to the clinical task and procedural technique [6]. By this, patients are protected against unnecessary radiation exposure. In Europe, national DRLs, anatomical DRLs, mostly, are defined for 13 different DX projections, on average [8]. The outcome of this survey underlined the need to establish clinical DRLs for a more extensive list of regularly performed DX procedures for which (national) DRLs have not yet been defined. It was found that most systems do not comply with at least one currently defined DRL. In some cases, the deviation was quite large (e.g., system 8 by 288% for knee LAT), even though the responsible medical physicists had emphasized their commitment to optimizing procedures. The reason for dose outliers and dose variations may be the lack of knowledge about the doses expected under good clinical practice or the lower attention to procedures for which DRLs have not yet been defined. Furthermore, the technical parameters of procedures of the extremities are usually set manually by the radiographer instead of using the automatic exposure control to adapt the dose to patient size, which can further increase dose variation.

As participants of the study were asked to investigate image and protocol parameters in cases of high or low *P*_KA_ values, it was found that dose outliers and large interquartile ratios could be often ascribed to non-optimized protocol settings, the use of non-optimized exposed areas, or a non-correct object centering. In one case, technical limitations of an older detector system (unit 56) were the reason for relatively large doses.

To the best of our knowledge, only a few clinical DRLs have been defined for DX in a few European countries, so far [8]. A clinical DRL is an investigation level for similar, or even the same, clinical tasks, the same anatomical location, and the same procedural technique. Within the anatomical location, the clinical task and/or the procedural technique can differ. If doses significantly differ, separate clinical DRLs should be defined [6]. For instance, task (Table 1), exposed area (C3–C7 vs. C1–C3), technique (open mouth), and *P*_KA_ (10 cGycm^2^ vs. 7 cGycm^2^, Table 2) differ between the examination of the cervical spine AP and the examination of the dens axis, respectively. Therefore, it is recommended to define separate DRLs for these projections. This recommendation also applies to the exposure of the entire spine with a *P*_KA_ of 289 cGycm^2^ for the AP-view (Table 2) that is considerably smaller than the sum of national DRLs of corresponding sections of the spine (e.g., Swiss DRL for the DX examination of the lumbar spine AP alone is 235 cGycm^2^, [20]). This dose difference results from different clinical indications. While the geometrical assessment of the entire spine requires relatively low-dose values, a higher value (e.g., Swiss DRL for lumbar spine) is used for assessing herniated discs or osseous changes of the spine, such as osteoporosis [4]. In the chest region, clinical DRLs were defined for the examinations of the ribs. The 3rd quartile of median values of ribs AP (48 cGycm^2^) is significantly by a factor of 3.2 larger than the national DRLs for the examination of the chest posterior-anterior (PA) (e.g., Austrian DRL 15 cGycm^2^ [21]). This dose discrepancy results from different tube voltages (73 kV vs. 125 kV for the radiographs of the ribs and chest, respectively). The lower voltages used for the radiographs of ribs are used for high-contrast imaging of bones. This underlines the importance of the definition of separate DRLs for different clinical indications. As with CT and IR, different DX procedures in the same anatomical region could require significantly different dose values due to different medical questions and different required image quality. Therefore, the definition of purely anatomical DRLs is no longer sufficient, and there is no reason not to extend the concept of clinical DRLs to DX procedures.

Usually, the label of a radiograph or the protocol name was used to assign individual radiographs to the reference projections. For clinical DRLs, this label should allow a clear separation of exposures concerning the CAP approach. This requirement was not always fulfilled in this study: The reason for the increased doses used for knee LAT at systems 7–9 was the use of an increased exposed area regardless of the clinical task. An increased area is justified, for example, in preoperative examinations for planning endoprosthesis surgeries. System 73 was used in the emergency room of a hospital where many radiographs in difficult conditions were performed (patients’ physical limitations and under time pressure with an urgent need for the images to make a clinical decision). This explains the increased interquartile ratio of 4.3 for the cervical spine LAT for this system. A strict separation of procedures with different tasks (and significantly different doses) would have avoided this large intra-system dose variation. Furthermore, some of the large dose variations indicate the need to improve the differentiation of some procedures with potentially different clinical tasks: for the exam of the ribs, for instance, often the half chest is exposed (hemithorax). In this study, however, it was not considered that the exposed area of the projections could be reduced (in case of more localized pain), resulting in a smaller *P*_KA_. This resulted in a relatively large maximum intra-system dose discrepancy of 11. For the examination of the entire spine, the pelvis or cervical spine does not have to be necessarily included in the radiograph, which was also not considered when defining the list with the reference projections (Table 1).

Users from different countries participated in the survey, and as a result, a more robust database could be obtained. This international approach also complies with a request of the ICRP and the EC to harmonize the exposure practice of facilities even across country borders [6, 8]. The discussions about the results encouraged the exchange of knowledge and experiences among the participants, including familiarization with national healthcare system-related differences. Nevertheless, based on the submitted dose-related data, the initial assumption was confirmed, that there are no significant differences in the exposure practice in DX in facilities of different (Central) European countries. The only significant differences found resulted from unilateral efforts in one country’s facilities to reduce the dose for specific procedures and did not originate from significantly different technical standards or different requirements of image quality.

For data acquisition and computation, locally installed DMS should be used and could reduce workload compared to previous surveys [14]. For examinations where 2–3 single exposures were combined (stitched radiographs), it was found important to provide *P*_KA_ values for the total procedure (at best, in the DICOM structured report), rather than of the single exposures.

We take our study as an indication that the technical equipment and exposure practices in X-ray facilities in most European countries differ little or not at all. Therefore, the DRL found offer users beyond the participating countries of the study, Austria, Germany, Italy and Switzerland, an orientation for optimizing the corresponding DX procedures. The new DRLs will be made available to the relevant authorities in Austria, Germany, Italy and Switzerland. In Germany, they form the basis for the data acquisition for future national DRLs.

Our study has several limitations. Firstly, DRLs were defined for radiographs whose radiation risk for damaging effects is small (usually, the effective dose is smaller than 1 mSv). However, DX is by far the most common X-ray procedure in Austria (compared with all procedures, the relative frequency is 88.4%), Germany (86.9%), Italy (85.8%), and Switzerland (92.0%). Their relative contribution to the overall population dose is 17.8%, 14.6%, 14.2%, and 15.9%, respectively [22]. As underlined by the ICRP, optimization of conventional radiographs, including examinations of extremities, is necessary [6]. Secondly, the image quality achieved was not evaluated. Therefore, there is the risk that dose values were not adapted to the specific clinical task. Thirdly, pediatric patients’ data were not enrolled. The optimization of X-ray procedures for children is an important part of mitigating long-term consequences. Fourthly, for each of the reference procedures, the participants were asked to report the quartiles computed from at least *10* representative *P*_KA_. Although this requirement corresponds to the German and the European approach to determining DRLs [14, 23], it contradicts the ICRP [6] that recommended a database of 20 values at least. However, it must be underlined, that independent of the specified minimum requirements, more than 82% of all the dose-related data provided in this study is based on much more than 20 single dose-relevant parameters. Lastly, the DRLs derived in this study do not necessarily represent the exposure practice in the corresponding countries. The doses of the surveyed systems were routinely optimized by medical physicists which could also result in a systematic bias to smaller doses than usually applied. This bias could be further increased by the fact that 63% of the systems were produced by two manufacturers, only.

## Conclusion

In this international multicenter study, dose-relevant data from 85 DX systems from Austria, Germany, Italy, and Switzerland were obtained to define clinical DRLs for 24 DX projections. For dose data acquisition, quartiles and the mean of the dose distribution of *P*_KA_ were computed using the locally installed DMS. Dose values varied considerably both in single systems and between institutions. Therefore, there is a high potential for dose optimization and harmonization, e.g., by adapting protocol parameters to the anatomical region *and*, if the doses significantly differ, to the clinical task and/or procedural technique. In this context, clinical DRLs can help in the optimization of dose even for procedures in the low-dose range: they can serve as a baseline for users for comparison with local practice (as local DRL, [6]), clinical audits [24], for competent authorities to establish national DRLs in future and in reducing the overall population dose. This survey underlines the importance of stronger international cooperation among users of ionizing radiation, exchanging experiences and knowledge, establishing a larger database for the definition of DRLs, and harmonizing the exposure practice across state borders.

## Abbreviations

ALARA: As low as reasonably achievable
AP: Anterior-posterior
APT: Working Group of Physics and Technology, Subgroup of the German Roentgen society
DMS: Dose management system
DRL: Diagnostic reference level
DX: Plain radiography/radiographic
EC: European Commission
ICRP: International Commission on Radiation Protection
LAT: Lateral
OBL: Oblique
PA: Posterior-anterior
*P*_KA_: Air-kerma-area product
TAN: Tangential
